# Gait pattern analysis in the home environment as a key factor for the reliable assessment of shunt responsiveness in patients with idiopathic normal pressure hydrocephalus

**DOI:** 10.3389/fneur.2023.1126298

**Published:** 2023-04-04

**Authors:** Sandra Fernandes Dias, Christina Graf, Elisabeth Jehli, Markus Florian Oertel, Julia Mahler, Marianne Schmid Daners, Lennart Henning Stieglitz

**Affiliations:** ^1^Department of Neurosurgery, University Hospital of Zurich, Zurich, Switzerland; ^2^Product Development Group Zurich, Department of Mechanical and Process Engineering, Eidgenoessische Technische Hochschule Zurich, Zurich, Switzerland; ^3^Translational Research Center, University Hospital of Psychiatry and Psychotherapy, University of Bern, Bern, Switzerland

**Keywords:** gait pattern, idiopathic normal pressure hydrocephalus (iNPH), gait disturbance, ventriculo-peritoneal shunt (VP-shunt), outcomes

## Abstract

**Background:**

The identification of patients with gait disturbance associated with idiopathic normal pressure hydrocephalus (iNPH) is challenging. This is due to the multifactorial causes of gait disturbance in elderly people and the single moment examination of laboratory tests.

**Objective:**

We aimed to assess whether the use of gait sensors in a patient's home environment could help establish a reliable diagnostic tool to identify patients with iNPH by differentiating them from elderly healthy controls (EHC).

**Methods:**

Five wearable inertial measurement units were used in 11 patients with iNPH and 20 matched EHCs. Data were collected in the home environment for 72 h. Fifteen spatio-temporal gait parameters were analyzed. Patients were examined preoperatively and postoperatively. We performed an iNPH sub-group analysis to assess differences between responders vs. non-responders. We aimed to identify parameters that are able to predict a reliable response to VP-shunt placement.

**Results:**

Nine gait parameters significantly differ between EHC and patients with iNPH preoperatively. Postoperatively, patients with iNPH showed an improvement in the swing phase (*p* = 0.042), and compared to the EHC group, there was no significant difference regarding the cadence and traveled arm distance. Patients with a good VP-shunt response (NPH recovery rate of ≥5) significantly differ from the non-responders regarding cycle time, cycle time deviation, number of steps, gait velocity, straight length, stance phase, and stance to swing ratio. A receiver operating characteristic analysis showed good sensitivity for a preoperative stride length of ≥0.44 m and gait velocity of ≥0.39 m/s.

**Conclusion:**

There was a significant difference in 60% of the analyzed gait parameters between EHC and patients with iNPH, with a clear improvement toward the normalization of the cadence and traveled arm distance postoperatively, and a clear improvement of the swing phase. Patients with iNPH with a good response to VP-shunt significantly differ from the non-responders with an ameliorated gait pattern.

## Introduction

The first description and correlation between gait impairment and hydrocephalus were made in 1965 by Hakim and Adam (1). The etiology of idiopathic normal pressure hydrocephalus (iNPH) has not yet been entirely understood (2–5). In elderly patients, other conditions such as spinal canal stenosis, Parkinson's disease, and polyneuropathy may influence the gait negatively.

Thus far, the diagnosis of NPH has been performed based on clinical symptoms, radiological findings, responsiveness to cerebrospinal fluid (CSF) tap tests, and gait analysis in laboratory settings (6). Long-term follow-up examination has shown a clinical improvement of 70–90% in patients with iNPH after ventriculo-peritoneal (VP)-shunt placement, particularly in gait (6, 7). From the Hakim triad, gait disturbance seems to occur in ~91% of all patients with iNPH (8, 9).

The typical walking disorder of a patient with iNPH is characterized by short-stride length, wide-stride width, outwardly rotated feet, low-stride height (“ironing” walk), and multiple steps required for turnaround and flailing arms. A recent study performed on a pressure sensitive carpet confirmed the hypokinetic gait characteristics of iNPH patients with reduced walking speeds, reduced stride lengths, and confirmed the requirement for more broad-based support (10). Nevertheless, in the clinical/laboratory setting, pathological gait characteristics may not be apparent due to the unnatural situation and the patient's efforts to perform well in front of the medical staff (11–13). Therefore, new efforts and methodological approaches for gait analysis have been exploited to better understand and characterize the walking pattern present in specific neurologic disorders, including iNPH (13–15). The ambulatory center of mass movement studies showed that patients with iNPH tend to have decreased gait velocities, increased step numbers, and increased step times, when compared to healthy controls (16).

Surgery seems to be an effective treatment, with up to 80% of patients showing clinical improvement, but only 20% of affected patients underwent surgery (17). After VP-shunt placement, patients experience to some degree an improvement in gait, better cognition, and relief from urinary incontinence. Montreal Cognitive Assessment (MoCA) is used often as a postoperative indicator of increased cognitive function (18). The Timed Up and Go (TUG) and 18-m walking tests can be used preoperatively after a CSF tap test, as well as postoperatively for the assessment of the gait apraxia in terms of responsiveness to VP-shunt. (19, 20).

A recent study, using portable inertial measurement units (IMUs), showed the benefit of gait assessment in the patient's normal environment in comparison with the single picture gait analysis obtained in a laboratory (21). The idea behind using IMUs for iNPH examination and diagnostics is to identify characteristics of this typical walking pattern in a home environment and to allow an automatized identification of patients with iNPH. Using this IMUs technology, which records accelerometry and gyroscopy, we aimed to characterize the walking pattern of patients with iNPH in the home environment preoperatively and postoperatively after VP-shunt placement while differentiating them from EHCs. Through the postoperative gait analysis, we aimed to identify “responders” and “not responders” to VP-shunt.

## Methods

### Subjects

A total of 11 patients (six female patients) were diagnosed with iNPH, and a control group of 20 EHCs (10 female subjects) was included in this study. The patients with iNPH were matched to the EHCs according to sex and age (Table 1). All patients and EHC gave their informed consent in the study prior to participation. The study was approved by the Cantonal Ethics Committee Zurich (BASEC-No. 2018-00051) and Swissmedic (102597735).

**Table 1:** Demographic and anthropometric data of patients suffering from idiopathic normal pressure hydrocephalus (iNPH) and of the elderly healthy control (EHC) group.

**Group**	**Number of subjects**	**Female/male ratio**	**Age (years)**	**Age matching (years)**			**Height (cm)**	**Weight (kg)**
				**60–69**	**70–79**	**80–89**		
iNPH	11	6/5	77 (±6.7)	3 (27.3%)	3 (27.3%)	5 (45.4%)	169.0 (±11.3)	76.6 (±10.3)
EHC	20	10/10	74.5 (±8.6)	6 (30%)	7 (35%)	7 (35%)	171.4 (±9.7)	70.7 (±12.1)

All values for age, height, and weight are means with standard deviation in brackets. Frequency-based age matching is given in absolute frequency with percentage (%) in brackets. The age differences of the matched pair did not exceed a maximum of 6 years.

### Inclusion criteria

#### Idiopathic normal pressure hydrocephalus

The planned implantation of a VP-shunt for the treatment of iNPH fulfilling all clinical criteria was mandatory for all patients. Eligibility for surgery was considered by means of a lumbar tap test of at least 35 mL CSF, while an improvement of ≥20% in walking speed or ≥13% endurance needed to be achieved (22). All patients were medically examined. Patients presenting orthopedic disorders unable to walk or with additional neurologic deficits were excluded. In addition to the presence of Hakim's triad, radiological criteria with Evan's index of ≥0.3, the presence of a DESH sign, and an acute callosal angle (measured on the coronal layer at the level of the posterior commissure) were evaluated and taken into account when establishing the diagnosis of iNPH. The Kiefer scale score was obtained preoperatively and postoperatively, at the time of the examination (23). Based on this, the NPH-RR [preoperative Kiefer Scale (KS) score - postoperative KS score]/(preoperative KS score × 10) was calculated (24). A NPH recovery rate (NPH-RR) of ≥5 means a very good response to VP-shunt treatment (23).

#### Healthy elderly controls

Inclusion criteria for EHC included age ranging from 60 to 100 years and no clinical suspicion for iNPH or any other movement disorder. EHCs had no chronic illnesses that could interfere with the gait (e.g., diabetes) and were not on routine medication.

### Clinical assessment and experimental protocol

The clinical assessment of patients with iNPH was performed at the department of neurosurgery in the University Hospital, Zurich. All patients received a VP-shunt as treatment. In 10 patients, a programmable Codman Hakim valve (initial opening pressure of 160 mmH_2_O) with an anti-siphon device was implanted; and one patient received an M-Blue Plus (setting 5/30 cmH_2_O) valve. After surgery, all patients were discharged. No prescription for rehabilitation or physiotherapy was issued on a standard basis. The preoperative gait analysis was done in the previous week before the surgical intervention. The postoperative measurements were planned between the 3rd and 6th months after surgery. Cognition and urine continence were assessed postoperatively.

**Figure 1:**
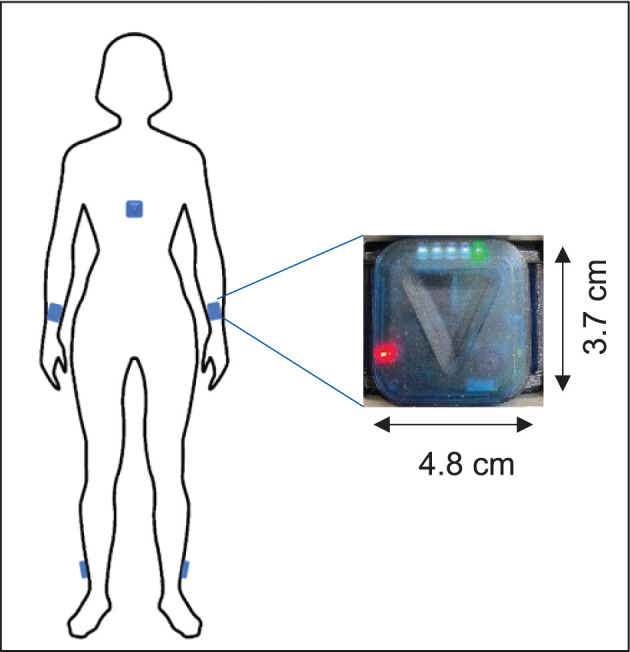
Illustration of the sensors' placement on the body.

Five ZurichMOVE sensors (25) were attached with a kinesiology tape to both ankles, wrists, and chest (Figure 1). Patients wore the sensors permanently for 72 h with the freedom of movement. To estimate the step width, which is needed for calibration, the distance of both feet while standing still in a normal and broad stand was recorded in a clinical setting. A supervised 10-m walking test at the hospital was carried out by all patients and subjects at their own speed. This distance was first walked without any additional specifications and then later with a defined step width (heel-to-heel) of 35 cm. Finally, a 180° turnaround was completed three times while the needed number of steps was counted. Primary endpoints described the IMU recordings and several walking parameters (11).

### Gait analysis

Fifteen temporal and spatial gait parameters were identified as characteristics of pathological gait in patients with iNPH (6, 15, 26). The algorithm used to extract the data from the wearable IMU sensors was developed by Renggli et al. (11). First, a step detection for every gait cycle by identifying the events of flat-foot, toe-off, and heel-strike was created. Second, all parameters were evaluated for each gait cycle, and the respective mean values and standard deviations were calculated. Details about the calculation and estimation methods can be found in the mentioned publication (11). We focused our gait analysis based on the parameters, in which a significant difference between the laboratory and home environment was observed.

### Statistical analysis

The data processing and statistical analysis were performed using MATLAB (MathWorks Inc., Natick, MA, USA) and SPSS (IBM SPSS Statistics, Chicago, IL, USA), respectively. First, a group analysis of gait parameters was performed using a multivariate analysis of variance (MANOVA) between EHC and patients with iNPH. Second, statistically significant values (*p*-value < 0.05) were further analyzed using Levene's test to assess the equality of variances and tests between subject effects. Finally, *post-hoc* tests (Bonferroni correction) were performed. Statistical significance was assumed when the *p*-value is <0.05.

Logistic regression was used to determine whether the preoperative gait parameters would be indicators for a better response to VP-shunt placement (NPH-RR ≥ 5). The results were expressed as relative risk (RR) with 95% confidence intervals (CIs). The receiver operating characteristics (ROC) analysis was performed using the pROC package (27).

## Results

The demographics and anthropographics of the 11 patients with iNPH and 20 EHCs are presented in Table 1. The radiological parameters of all patients are demonstrated in Table 2.

**Table 2:** Radiological data of patients suffering from idiopathic normal pressure hydrocephalus (iNPH) and respective NPH recovery rate (RR).

**Patient**	**Evans' index**	**DESH sign**	**Callosal angle (°)**	**NPH-RR**
1	0.33	Positive	90.4	10
2	0.3	Negative	89.1	0
3	0.36	Positive	96.1	10
4	0.3	Positive	87.8	10
5	0.41	Positive	71.2	10
6	0.36	Positive	75.6	5
7	0.38	Negative	91.2	6.3
8	0.36	Positive	66.5	4.5
9	0.4	Positive	61.5	8.5
10	0.38	Negative	90.1	0
11	0.38	Positive	90.7	4

**Figure 2:**
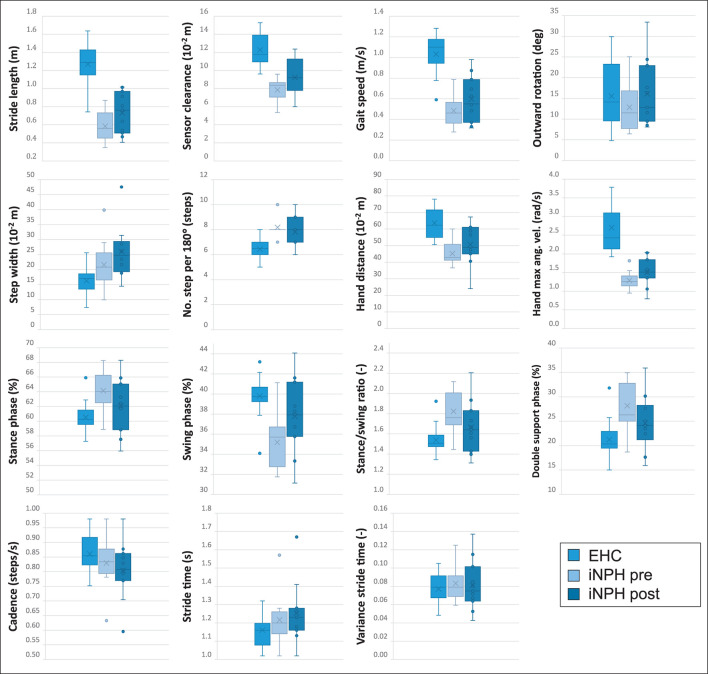
All illustrated boxplots indicate the comparison in a real-world environment between 20 elderly healthy controls (EHC) and 11 patients with normal pressure hydrocephalus (iNPH) preoperatively and postoperatively after ventriculo-peritoneal (VP)-shunt implantation. The absolute difference between the parameter's median values of every subject is indicated in each boxplot. Within each boxplot, the group's mean values are indicated as a cross, and outliers are indicated as dots. The upper and lower edges of the box define the interquartile range, and the whiskers indicate the extreme data points.

Overall, in terms of average daily activity detection, walking comprised 5.7% of the time in the EHC group, 2.5% in the iNPH postoperative, and 2.1% in the iNPH preoperative groups, respectively. The differences in gait characteristics between all groups (EHC, iNPH preoperatively, and postoperatively) during the 3-day analysis in a home environment are summarized in Figure 2. The statistical results are summarized in Table 3 and described in detail below.

**Table 3:** Spatial and temporal gait parameters for the evaluation of a 72-h monitoring using wearable inertial measurement unit (IMU) sensors.

**Parameter (symbol)**	**Unit**	**iNPH pre vs. EHC**	**iNPH post vs. EHC**	**iNPH pre vs. iNPH post**
		**MANOVA**		
Stride length (SL)	cm	**<0.001**	**<0.001**	0.157
Max foot clearance (FC_max_)	cm	**<0.001**	**<0.001**	0.052
Gait velocity (V_Gait_)	m/s	**<0.001**	**<0.001**	0.234
Foot outward rotation (Θ)	°	1.000	0.204	1.121
Step width (SW)	cm	**0.034**	**<0.001**	0.417
Steps per 180° turn (*n*_StepsTurning_)	–	**<0.001**	**<0.001**	1.000
Stance phase (*P*_Stance_)	% of gait cycle	0.432	0.054	1.000
Swing phase (*P*_Swing_)	% of gait cycle	0.304	0.583	**0.042**
Double limb support phase (PDL)	% of gait cycle	1.000	0.172	0.572
Stance to swing ratio (R_StanceToSwing_)	-	0.345	0.262	1.000
Cadence (*n*_Cycle_)	spm	**0.007**	0.137	0.799
Cycle time (T_Cycle_)	s	1.000	0.126	0.488
Cycle time deviation [dev(T_Cycle_)]	%	**<0.001**	**<0.001**	1.000
Arm swing amplitude (A_Swing, Arm_)	rad/s	**<0.001**	**<0.001**	1.000
Traveled arm distance (dist_Arm_)	cm	**0.007**	0.144	0.800

The statistical outcomes were made by using a multivariate analysis of variance (MANOVA) between EHC and patients with iNPH and thereafter between iNHP preoperatively and postoperatively. Statistically significant values (*p*-value < 0.05) were further analyzed using Levene's test to assess the equality of variances and tests between-subject effects. Bonferroni correction was used. Statistical significance was assumed when the *p*-value is < 0.05 (values in bold).

### EHC vs. patients with iNPH preoperative

A statistically significant difference between EHC and patients with iNPH preoperatively was observed in 9/15 (60%) of tested gait parameters, with an exception of foot outward rotation, stance phase, swing phase, double limb support phase, stance to swing ratio, and cycle time.

### EHC vs. patients with iNPH postoperative

After VP-shunt placement, a change toward ameliorated walking patterns was observed in patients with iNPH (Figure 2), particularly in terms of cadence and traveled arm distance, in which the preoperatively statistical significance was not observed anymore, with *p* = 0.137 and *p* = 0.144, respectively. Compared to the preoperative phase, differences in the foot outward rotation, stance phase, swing phase, double limb support phase, stance to swing ratio, and cycle time remained not statistically significant between groups.

### Patients with iNPH preoperative vs. postoperative

In the iNPH group, even though patients reported a subjective improvement in walking postoperatively, only the swing period (*p* = 0.042) was statistically significant (Table 3).

### Responders vs. non-responders to VP-shunt treatment

**Figure 3:**
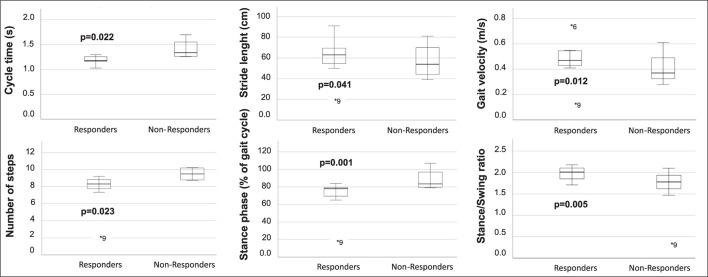
Boxplots of the gait parameters that were statistically significant between responders (NPH-RR ≥ 5) and non-responders to VP-shunt implantation. Respective *p*-values are stated in bold face. Each plot indicates the absolute difference between the parameter's median values for each group. Within each boxplot, the group's median value is indicated as a line, and outliers are indicated as dots (*).

**Figure 4:**
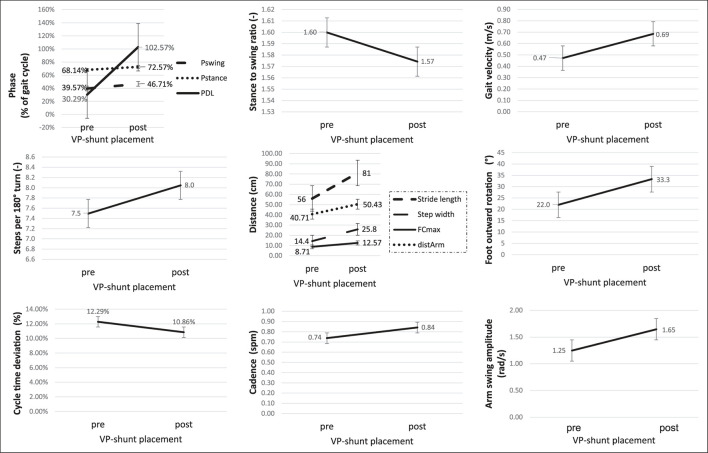
Graphic illustration of the changes in the gait parameters in the responder's group (NPH-RR ≥ 5) to VP-shunt implantation. Error bars represent standard errors. Fcmax, maximal foot clearance; distArm, traveled arm; Pswing, swing phase; Pstance, stance phase; PDL, double limb support phase.

A sub-group analysis of the gait parameters between responders (NPH-RR ≥ 5) and non-responders to VP-shunt showed a statistically significant difference between groups in terms of gait velocity (*p* = 0.012), number of steps (*p* = 0.023), cycle time (*p* = 0.004), stride length (*p* = 0.041), stance to swing ratio (*p* = 0.005), stance phase (*p* = 0.001), and cycle time (*p* = 0.012) (Figure 3). These results corroborated the overall gait improvement observed (Figure 4).

The presence of a positive DESH sign (RR 8.0; 95% CI 1.28–50.04) favors a better response to VP-shunt placement. An Evans Index value of >0.35 (RR 1.07; 95% CI 0.41–2.80) or a callosal angle of <75° (RR 0.43; 95% CI 0.18–1.01) did not seem to be prone to a better response to VP-shunt treatment. Patients with a callosal angle of 75° were more likely to have a good response to VP-shunt placement (RR 2.33; 95% CI 0.99–5.49).

**Figure 5:**
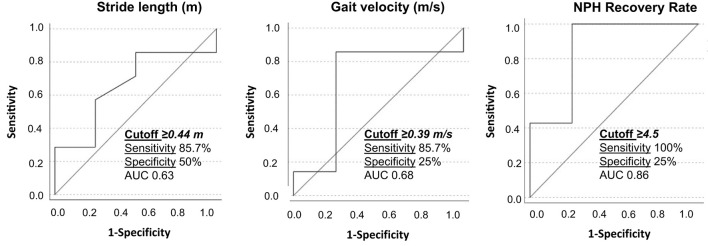
Receiver operating characteristic curves for stride length, gait velocity, and NPH recovery rate between responders and non-responders to VP-shunt implantation. The respective cutoff values, sensitivity, specificity, and area under the curve (AUC) are given within the graphics.

A ROC analysis was performed based on the obtained statistically significant gait parameters. The following cutoffs were obtained as possible predictors for good VP-shunt responsiveness: a stride length of ≥0.44 m and a gait velocity of ≥0.39 m/ with a sensitivity of 85.7% (Figure 5).

### Adverse events

No complications related to the use of the sensors (discomfort and bruises) were reported.

## Discussion

This study presents the data of gait patterns investigation in the home environment, between EHCs and patients with iNPH preoperatively and postoperatively, for 3 days without restrictions on daily activities. The gait pattern analysis of patients with iNPH has been included in some studies (10, 14, 15, 20), but these were performed in laboratory and medical environments. In addition, gait disturbances occurring from other neurologic disorders can also be present (28). Comparing the walking tests (in laboratory settings) of patients with Parkinson's disease and iNPH, it was found that patients with iNPH usually present with more severe gait dysfunction, which improves after lumbar puncture (14).

The use of smartphone applications has emerged in the last few years to help perform gait pattern assessments in the home environment (29). One of the main applications so far is in the gait analysis of patients with Parkinson's disease. The applications use accelerometer and gyroscope data to assess balance and gait and to detect the freezing of gait patterns (30). The use of such technology with patients with iNPH has been reported in two patients so far (31). A recent study investigated the walking pattern of healthy subjects and patients with iNPH in a controlled and real-world environment through the use of wearable gait assessment devices (21). The authors concluded that the measurements taken in the home environment showed better discrimination of the patients with iNPH compared with the laboratory environment (21). Based on these results, we aimed to assess whether, in a home environment, the use of these sensors could help establish a reliable diagnostic tool to identify patients with iNPH. Our study showed a statistically significant difference for 60% of the gait parameters tested between EHC and iNPH groups, which supports the argument that gait tests performed in a home environment are more reliable for the diagnosis of iNPH than in a controlled laboratory environment (21).

Postoperatively, we assessed whether the gait pattern of patients with iNPH was normalized or at least got closer to the EHC. The cadence and traveled arm distance of patients with iNPH after VP-shunt placement did not differ anymore from the EHC, reflecting a significant change toward gait normalization. Foot outward rotation was thought to be one typical feature of iNPH (2). Interestingly in our study, no significant difference was observed between EHC and iNPH groups. These results show that in the end, the foot's outward rotation might be more intrinsic to the walking characteristics of each subject than a pathological gait marker. The swing phase (during which the raised leg is moved forward) occupies 40% of the gait cycle and is commanded by the big muscle groups of the leg (e.g., *M. rectus* femoris and *M. quadriceps* femoris). This parameter did not significantly diverge between the iNPH and EHC groups, which could be explained through the inter-individual homogeneity obtained by the age matching (32). However, a significant improvement was observed postoperatively in the iNPH group. The fact that the postoperative tests were performed within 6 months after surgery may have contributed to seeing the difference.

Previous studies aimed to assess the recovery process of gait disturbances after VP-shunt surgery in patients with iNPH using several tests in a controlled setting (20, 33, 34). For the first time, we aimed to get a more reliable evaluation concerning the response of patients with iNPH to VP-shunt placement by comparing the gait pattern of patients with iNPH preoperatively and postoperatively in the home environment. Our results indicate that after VP-shunt placement, patients experience an improvement in gait performance. A statistically significant difference in the swing period (*p* = 0.042) shows that patients with iNPH walk with better stability after surgery. The walking speed did not significantly change, which could be explained by the heterogeneity of responses to VP-shunt placement within the iNPH group but also by the small number of patients included.

For the first time, we identified the patients with iNPH with a good response to VP-shunt (NPH-RR ≥ 5) (7) and analyzed their gait parameters separately. A clear improvement of the majority of the gait parameters could be observed in the responders' group, with the parameters gait velocity, stance to swing ratio, stance phase, number of steps, stride length, and cycle time reaching statistical significance in comparison to the non-responders. These results objectively support the clinical observation that a specific group of patients with iNPH with a good NPH-RR undergoes a significant gait improvement postoperatively. Nevertheless, these results should be interpreted with caution due to the small sample size. This explains why the area under the curve (AUC) of the ROC analysis showed only modest accuracy. The cutoff values obtained for the stride length and gait velocity might give us an idea preoperatively of which patients could show good responsiveness to VP-shunt placement. A larger sample of patients is needed to confirm and support these results.

Worldwide recognized as a marker of ventriculomegaly (35), an Evans Index value of >0.35 was not associated with a better response to VP-shunt placement (RR 1.07). The DESH sign has been established as an important radiological feature for the diagnosis of NPH (8, 36). Our results support this evidence as patients with a positive DESH sign were more likely (RR 8.0) to have an NPH-RR of≥5. Interestingly, even though in the literature a preoperative callosal angle of 63° has been shown to have higher prognostic accuracy to VP-shunt response (37), our data showed that patients with a callosal angle > 75° seemed to have a higher chance of having an NPH-RR of≥5 (RR 2.33), compared to patients with a callosal angle of <75° (RR 0.43). This could be related to the time of diagnosis, during which this patient's cohort might have been earlier diagnosed, therefore presenting with a wider angle. Further data from clinical studies are needed for a better understanding of these findings.

A significant proportion of patients with iNPH still do not respond properly to VP-shunt placement or even deteriorate (38). Potential contributors for non-responders could be older age, and the presence of comorbid neurologic conditions that is commonly associated with age (39). This may explain why patients with iNPH still have a pathological walking pattern compared to the EHC.

Overall, in this study, we observed that a clear pathological gait pattern can be reliably identified in the home environment when comparing EHCs and patients with iNPH preoperatively and postoperatively. After VP-shunt placement, an improvement in cadence and traveled arm distance toward normalization was observed. Furthermore, patients with iNPH showed significantly better walking stability postoperatively. Within the iNPH group, the responders' cohort benefited clearly from VP-shunt placement, showing a distinguished improvement in several gait patterns compared to the non-responders.

### Limitations

The results we obtained were based on a small sample size of patients with iNPH, which is the main limitation of this study. Due to the nature of the study, we could only include patients that were capable of walking.

Concerning data acquisition, in the case where a sensor gets dislocated in the home environment, the data acquisition could become impaired. To overcome this issue, we attached the sensors to the patient's skin with watertight drapes. Patients were instructed not to reposition a loose sensor themselves.

Even though an attempt was done to find cutoff values for preoperative variables of good responsiveness to VP-shunt placement, the ROC-AUC resulted in only a very narrow accuracy, which could be related to the small sample size. A bigger number of patients with iNPH leading to a higher amount of data as well as the use of machine learning algorithms will enable us to overcome this limitation.

### Future

We continue to assess whether, in a real-world environment, the use of the sensors could help to establish an automatic diagnostic tool for iNPH. Therefore, we will keep recruiting more patients. A sub-analysis of the sensors' data, where we will use only the two feet sensors and the chest reference, will help to evaluate whether a reduction of the number of sensors will lead to comparable results. We aim to develop a machine learning-based algorithm that is able to distinguish EHC and patients with iNPH and enables to predict which patients with iNPH will respond to VP-shunt treatment.

## Conclusion

A clear difference in gait patterns between EHC and patients with iNPH preoperatively and postoperatively has been identified based on walking assessment in the home environment. A statistically significant difference was observed in 60% of the analyzed gait parameters between EHC and patients with iNPH preoperatively. After VP-shunt placement, patients with iNPH presented a cadence and traveled arm distances similar to the EHCs. Compared to the preoperative phase, a statistically significant improvement in the swing phase (*p* = 0.042) was achieved by the iNPH group. This illustrates an objective improvement in walking stability. This walking pattern amelioration was even more evident in the responder's cohort (NPH-RR ≥ 5), with a statistical significance for gait velocity, stance to swing ratio, stance phase, number of steps, stride length, and cycle time. A ROC analysis revealed the cutoff stride length of ≥0.44 m and gait velocity of ≥0.39 m/s as predictors for good VP-shunt responsiveness. Overall, this study suggests that there is a sub-group of patients with iNPH that represents the ideal surgical candidates and can benefit the most from VP-shunt placement.

## Data availability statement

The datasets of the study are not publicly available because test subjects and patients were guaranteed that their data will be anonymously used for this study only and not passed to extern instances. Requests to access the datasets should be directed to LS, lennart.stieglitz@usz.ch.

## Ethics statement

The studies involving human participants were reviewed and approved by the Cantonal Ethics Committee Zurich (BASEC-No. 2018-00051) and Swissmedic (102597735). The patients/participants provided their written informed consent to participate in this study.

## Author contributions

SD and CG: data collection and analysis, statistical analysis, results discussion, manuscript elaboration, and revision. EJ: statistical analysis, result discussion, and manuscript revision. MO and JM: result discussion and manuscript revision. MSD and LS: study supervision, result discussion, and manuscript revision. All authors agreed to be accountable for the content of the study. All authors contributed to the article and approved the submitted version.
